# Response: Commentary: Chronic SSRI Stimulation of Astrocytic 5-HT_2B_ Receptors Change Multiple Gene Expressions/Editings and Metabolism of Glutamate, Glucose and Glycogen: A Potential Paradigm Shift

**DOI:** 10.3389/fnbeh.2015.00308

**Published:** 2015-11-13

**Authors:** Leif Hertz, Douglas L. Rothman, Baoman Li, Liang Peng

**Affiliations:** ^1^Laboratory of Brain Metabolic Diseases, Institute of Metabolic Disease Research and Drug Development, China Medical UniversityShenyang, China; ^2^Magnetic Resonance Research Center, Diagnostic Radiology and Biomedical Engineering, Yale UniversityNew Haven, CT, USA

**Keywords:** 5-HT_2B_ receptor, serotonin transporter, SSRIs, astrocytic transactivation, PLA2

Hertz et al. (2015) provided data indicating that the indisputable SERT inhibition caused by SSRIs is an epiphenomenon. Instead we pointed toward acute and chronic effects of fluoxetine on 5-HT_2B_ receptor stimulation in astrocytes (in culture or freshly isolated from brains of mice treated with fluoxetine for 14 days). Stimulation by fluoxetine of the astrocytic 5-HT_2B_ receptor causes a multitude of effects that in astrocyte cultures could be prevented by drug- or siRNA-induced 5-HT_2B_ receptor inhibition (Figure 1). This stimulation activates complex signaling pathways, including EGF receptor transactivation, a signal pathway in which a G-protein-coupled receptor (GPCR) signal leads to release of a growth factor, which activates the EGF receptor-tyrosinekinase in the same or adjacent cells. Astrocytes express a number of GPCRs and play key roles in brain function (Hansson and Rönnbäck, 2004; Fields, 2009). The autocrine effects of growth factor release may regulate gene expression and alter cell functions in the astrocytes themselves and the paracrine effects provide opportunities for effects on their neuronal neighbors. Our studies in mice treated with fluoxetine for 2 weeks showed multiple gene upregulations and editings (Li et al., 2012; Hertz et al., 2015), which altered the function of the gene product in kainate receptors, 5-HT_2_ receptors, phospholipase cPLA2, a Ca^2+^ L-channel gene and nucleoside transporter genes. These changes occurred mainly in astrocytes but some were neuronal. They occurred together with effects on metabolism of glucose and glycogen and turnover of glutamate and GABA, consistent with evidence of increased glutamatergic activity and decreased GABA-ergic activity in patients suffering from major depression, which are reverted by successful therapy; similarly, glucose metabolism is decreased in depressed patients and increases following treatment (Hertz et al., 2015).

**Figure 1:**
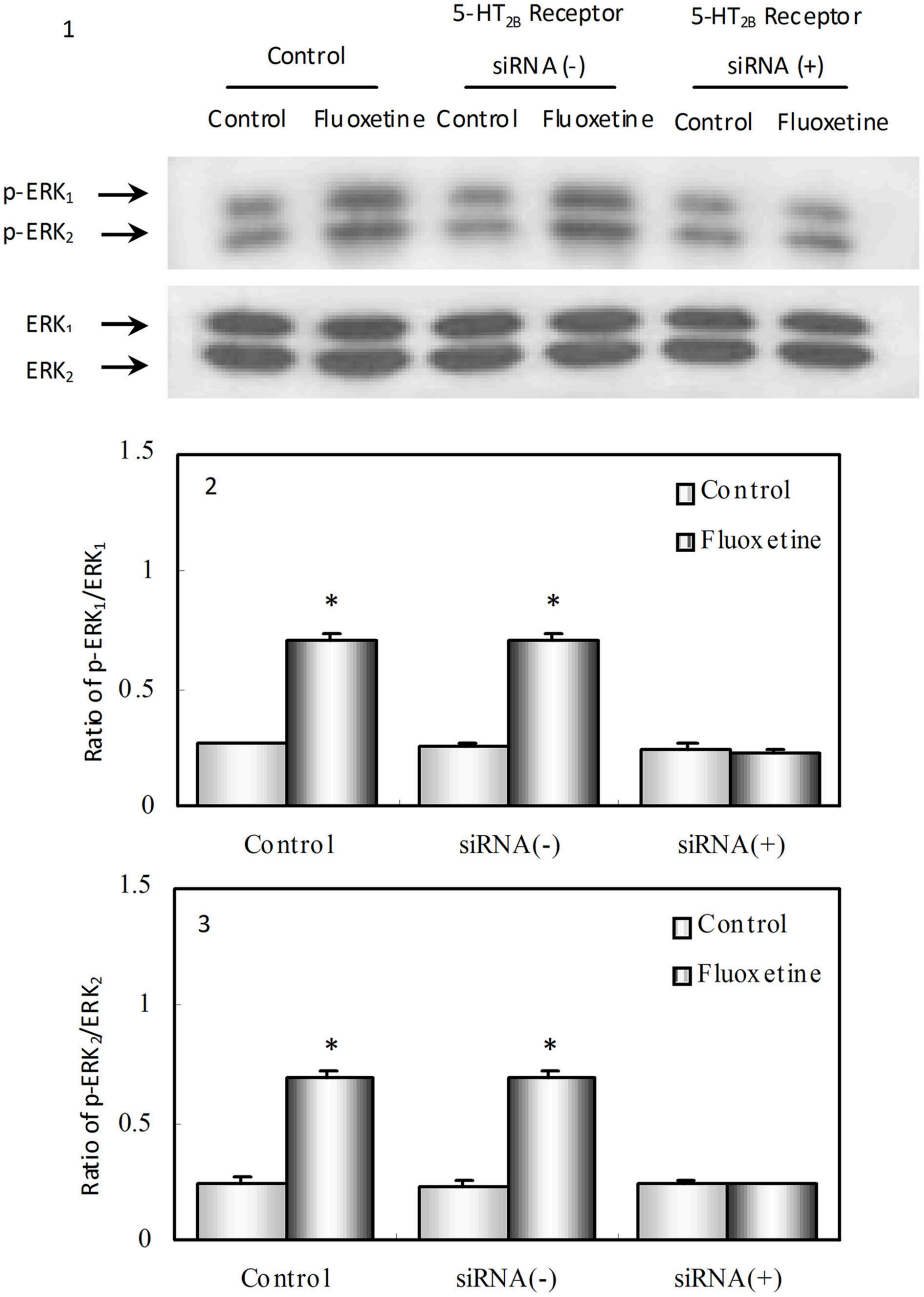
**Phosphorylation of ERK_1∕2,_ a known target of 5-HT_2B_ receptor stimulation in astrocytes**. After treatment with phosphate-buffered saline, PBS (Control), transfection solution without siRNA (siRNA (−)) or siRNA specific to 5-HT_2B_ receptor [siRNA (+)], culturing of well differentiated astrocytes (at least 3 weeks in culture and treated with dibutyryl cAMP [dBcAMP]), was continued for another week. Since 5-HT_2B_ receptor expression in the cultures had been normal until the exposure to siRNA the result indicates an effect on mature astrocytes. This contrasts the non-conditional gene knockouts of intact mice used by Diaz et al. (2012), where effects during development cannot be ruled out. For the phosphorylation experiments, the cells were incubated for 20 min in serum-free medium in the absence of any drug (Control) or in the presence of 10 μM fluoxetine. (1) Immunoblot showing gene expression of phosphorylated and non-phosphorylated ERK 1 and 2 (p-ERK_1_ and ERK_1_ and p-ERK_2_ and ERK_2_) from representative experiments. Similar results were obtained in three independent experiments. Average ERK phosphorylation was quantitated as ratios between p-ERK_1_ and ERK_1_ (2) and between p-ERK_2_ and ERK_2_ (3). SEM values are indicated by vertical bars. * Indicates statistically significant (*P* < 0.05) difference from all other groups for ERK_1_ and ERK_2_. (From Li et al., 2008).

A commentary by Banas et al. (2015) claim that we forgot important information provided by Diaz et al. (2012), including absence of antidepressant effects of fluoxetine or the 5-HT_2B_ agonist BW723C86 in mice lacking the serotonin transporter (SERT) or differentiated serotonergic neurons. They allege that these data rule out that the antidepressant effects of fluoxetine or BW723C86 could be SERT-independent (as claimed by us) and show that serotonergic neurons expressing SERT are necessary for the 5-HT_2B_ receptor effects exerted by fluoxetine (and other 5-HT_2B_ receptor agonists). They further remind us that Launay et al. (2006) in neuronal cultures from raphe nuclei demonstrated 5-HT_2B_ receptor-mediated control of SERT activity via 5-HT_2B_ receptor-promoted SERT phosphorylations. This should explain the finding by Diaz et al. (2012) that there are no antidepressant effects of either fluoxetine or BW723C86 in mice knocked-out for SERT or lacking differentiated serotonin neurons. These data should also rule out that the antidepressant effects of 5-HT_2B_ agonists, including fluoxetine, could be independent of SERT and explain the conclusion by the Maroteaux group (Diaz et al., 2012) that fluoxetine acts by 5-HT_2B_ receptor-mediated regulation of SERT in a cell autonomous manner.

We were well aware of the lack of antidepressant effect in SERT knock-outs described by Diaz et al. (2012) and of their reference to the paper by Launay et al. (2006), but did not comment on these points, because they are irrelevant. The lack of effect in the knock-outs does not prove any dependence on SERT, because Qu et al. (2005) showed that the direct DOI-mediated stimulation of 5-HT_2_ receptor activation of phospholipase A2 (cPLA2) and subsequent arachidonic acid release and metabolism seen in normal mice is abolished in mice lacking SERT. There is no information in the literature that DOI should interact with SERT. Although Qu et al. indicated DOI as a 5-HT_2A∕C_ agonist it also activates the 5-HT_2B_ receptor (Pineda-Farias et al., 2015). Stimulation of this receptor is the most likely reason for the response in wild-type animals, because fluoxetine acutely stimulates astrocytic 5-HT_2B_ receptors in cultured astrocytes (Li et al., 2008; Qiao et al., 2015) and after chronic administration (14 days) to mice upregulates this receptor in astrocytes but not in neurons (Li et al., 2012; Hertz et al., 2015). Moreover, cPLA2-mediated signal transduction is increased by acute fluoxetine administration in unanesthetized rats (Qu et al., 2003) and phospholipase A_2_ activity is potently stimulated by the SSRI sertraline in yeast (Rainey et al., 2010). Since direct stimulation with an agonist has no effect in these animals, the lack of fluoxetine effect in the knockouts does *not* prove fluoxetine dependence on SERT in adult brain.

With respect to the paper by Launay et al. it 2006 deals with extremely young cells. It does therefore not show that fluoxetine stimulates the 5-HT_2B_ receptor in mature individuals, because Homberg et al. (2011) and Sarkar et al. (2014) have shown that the serotonergic system in the immature brain functions in a completely different manner than in the mature brain.

Banas et al. also claim we changed results. However, the figure in Hertz et al. (2012) and in Kong et al. (2002) is—of course—the same and any reader has the opportunity to calculate *K*_*i*_ values based on the graph and the concentrations of mesulergine. The Zhang et al. (2010) paper, referred to in the Banas commentary clearly indicates the difference between SSRI affinity during acute and chronic treatment. There is no major difference between the acute affinity found by ourselves and others, and we are the only authors who have measured affinities for SSRIs in chronically treated cells. The suggestion that effects on muscarinic acetylcholine and histamine receptors or other monoamine transporters should explain our observations independently of “putative” direct agonist effects at 5-HT_2B_ receptors is invalidated by the observation by Li et al. (2008) that “ERK phosphorylation was abolished by SB204741, a universal 5-HT_2_ receptor antagonist, and in 5-HT_2B_ receptor-depleted cells (see Figure 1), but unaffected by 5-HT_2A_ or 5-HT_2C_ receptor antagonists.” Fluoxetine stimulation of astrocytic 5-HT_2B_ receptors was confirmed by Qiao et al. (2015). The “putative” receptors were demonstrated on both well-differentiated astrocyte cultures and freshly isolated astrocytes (Li et al., 2008, 2012; Peng et al., 2010; Zhang et al., 2010).

The commentary by Banas et al. has accordingly not altered our original conclusions but rather strengthened it.

## Conflict of interest statement

The authors declare that the research was conducted in the absence of any commercial or financial relationships that could be construed as a potential conflict of interest.
